# A Window of Opportunity: Targeting Cancer Endothelium to Enhance Immunotherapy

**DOI:** 10.3389/fimmu.2020.584723

**Published:** 2020-11-11

**Authors:** Gizem Duru, Marjolein van Egmond, Niels Heemskerk

**Affiliations:** ^1^ Amsterdam UMC, Vrije Universiteit Amsterdam, Department of Molecular Cell Biology and Immunology, Cancer Center Amsterdam, Amsterdam Infection & Immunity, Amsterdam, Netherlands; ^2^ Amsterdam UMC, Vrije Universiteit Amsterdam, Department of Surgery, Cancer Center Amsterdam, Amsterdam, Netherlands

**Keywords:** tumor angiogenesis, cancer endothelium, immune cell trafficking, vascular normalization, immunotherapy

## Abstract

Vascular abnormalities in tumors have a major impact on the immune microenvironment in tumors. The consequences of abnormal vasculature include increased hypoxia, acidosis, high intra-tumoral fluid pressure, and angiogenesis. This introduces an immunosuppressive microenvironment that alters immune cell maturation, activation, and trafficking, which supports tumor immune evasion and dissemination of tumor cells. Increasing data suggests that cancer endothelium is a major barrier for traveling leukocytes, ranging from a partial blockade resulting in a selective endothelial barrier, to a complete immune infiltration blockade associated with immune exclusion and immune desert cancer phenotypes. Failed immune cell trafficking as well as immunosuppression within the tumor microenvironment limits the efficacy of immunotherapeutic approaches. As such, targeting proteins with key roles in angiogenesis may potentially reduce immunosuppression and might restore infiltration of anti-tumor immune cells, creating a therapeutic window for successful immunotherapy. In this review, we provide a comprehensive overview of established as well as more controversial endothelial pathways that govern selective immune cell trafficking across cancer endothelium. Additionally, we discuss recent insights and strategies that target tumor vasculature in order to increase infiltration of cytotoxic immune cells during the therapeutic window of vascular normalization hereby improving the efficacy of immunotherapy.

## Introduction

Immunotherapy of cancer boost the host immune system to eradicate transformed cancerous cells. In the last decade a great variety of immunotherapeutic approaches emerged that transformed modern cancer treatment, including antibodies targeting immune inhibitory checkpoints (e.g., PD-1 and CTLA-4), monoclonal antibodies (mAbs) directed against tumor-associated antigens (TAA), anti-cancer vaccines, and chimeric antigen receptor T cells (CAR T cells). Novel immunotherapeutic approaches have expanded current treatment options tremendously, in particular for patients that have unresectable or metastatic solid tumors. Checkpoint blockade with PD-1 and CTLA-4 showed durable control of highly aggressive melanomas and has led to spectacular results showing rapid eradication of large metastatic tumors in response to one dose of Ipilimumab (CTLA-4) and Nivolumab (PD-1) (1, 2). As such, immunotherapy was acknowledged as breakthrough of the year 2017 by the journal Science and currently accepted as treatment option for many metastatic solid tumors including metastatic melanoma as well as lung cancer. However, immunotherapy does not help everyone. Approximately 50%–80% of patients that receive immunotherapy do not benefit from the treatment, and many patients experience severe side effects (3). Up to now multiple mechanisms have been described that can induce resistance to immunotherapy, underscoring the complexity and heterogeneity in immunotherapeutic responses between individuals.

Classifying tumors according their immunological status and localization of immune cells within the tumor supports the existence of three different cancer-immune phenotypes (4–6). Firstly, the immune inflamed phenotype is characterized by high infiltration of immune cells in the tumor. Secondly, the immune-excluded phenotype is characterized by the restriction of immune cells at the periphery of cancer nests (5, 6), or by confinement of immune cells in stromal regions of the tumor (7), which can also be present further towards the centre of the tumor (6, 8). Immune-excluded tumors may reflect the presence of inhibitory vascular growth factors (9), vascular barriers (10), inhibitory receptors on endothelium (11), might be induced by a specific chemokine state (12) or inhibition in stromal regions (7, 13). Lastly, the immune desert phenotype is characterized by limited neoantigens, tolerance induction and lack of appropriate T cell priming or activation (4–6), resulting in the absence of immune cells. While clinical responses to immunotherapy occur most often in patients with inflamed tumors, clinical responses in immune-excluded tumors are uncommon and not observed in tumors exhibiting the immune-desert phenotype (4, 14). Each immune phenotype is associated with specific underlying mechanisms that might limit immunotherapeutic responses. For instance, in immune deserts, a lack of sufficient neoantigens to activate cytotoxic T cells has been proposed while in inflamed tumors T cell exhaustion might be a major factor contributing to therapeutic insensitivity.

As an alternative explanation for the therapeutic variety in response rates to immunotherapy, we support the hypothesis that abnormal tumor vasculature fulfills a central position in selective immune cell trafficking, which in part regulatesthe localization of immune cells within the tumor site, reflecting the various immune phenotypes. In addition, abnormal tumor vasculature may have a key role in maintenance of immunosuppression in tumors, limiting immunotherapeutic responses (15–18). Dysfunctional tumor vessels give rise to hypoxia that in turn lowers pH, creates leaky vasculature, and impairs fluid drainage increasing interstitial fluid pressure, which altogether supports an unfavorable tumor microenvironment (TME) and cancer metastasis (19). These circumstances in tumors alter the functionality of many immune effector cells, leading to the release of immunosuppressive cytokines that disables immune effector functions rendering immunotherapy ineffective (20–24).

Equally important, angiogenic factors released in response to hypoxia interfere with immune cell trafficking (10, 11, 13, 25–29). The severity of impaired leukocyte entry into tumors ranges from a partial blockade resulting in a selective endothelial barrier in case of inflamed and in part in immune excluded tumors, to a complete immune infiltration blockade associated with tumors lacking immune infiltration. Several studies showed that the efficacy of immunotherapy was strongly related to tumor infiltrating leukocyte levels (30–34). The existence of non-inflamed tumors supports the idea that exclusion of immune cells from the TME plays an important role in cancer escape and resistance to immunotherapy.

Several lines of evidence have shown that vascular normalization using anti-angiogenesis agents could restore leukocyte recruitment (31–34) into the tumor microenvironment and relieve immunosuppression (32, 35, 36). Targeting dysfunctional tumor vasculature using antiangiogenic agents has therefore the potential to create a window of therapeutic opportunity in which immunotherapy could have a better outcome then used as a single therapeutic agent (15, 17, 37).

## How Abnormal Tumor Vasculature Contributes to Immunosuppression in Cancer

Early stage tumors are going through many cycles of growth and death. As nutrients and oxygen cannot reach cells in the tumor’s core, proliferation is followed by necrosis confining the size of the primary tumor to about 2–3 square millimeters (38). A tumor remains in this state until it acquires the ability to control its own growth through induction of angiogenesis. This event is called the angiogenic switch (39), which supports tumor progression in various ways. It improves supply of nutrients and oxygen and contributes to removal of toxic waste products (40). In addition, the connection to the vascular network facilitates infiltration of inflammatory immune cells into the tumor, increasing the complexity of the tumor microenvironment. Hypoxia as well as acquired mutations in proangiogenic growth factor pathways (e.g., loss of PTEN) have been implicated as key factors driving tumors into the angiogenic switch (17, 41–43).

**Figure 1:**
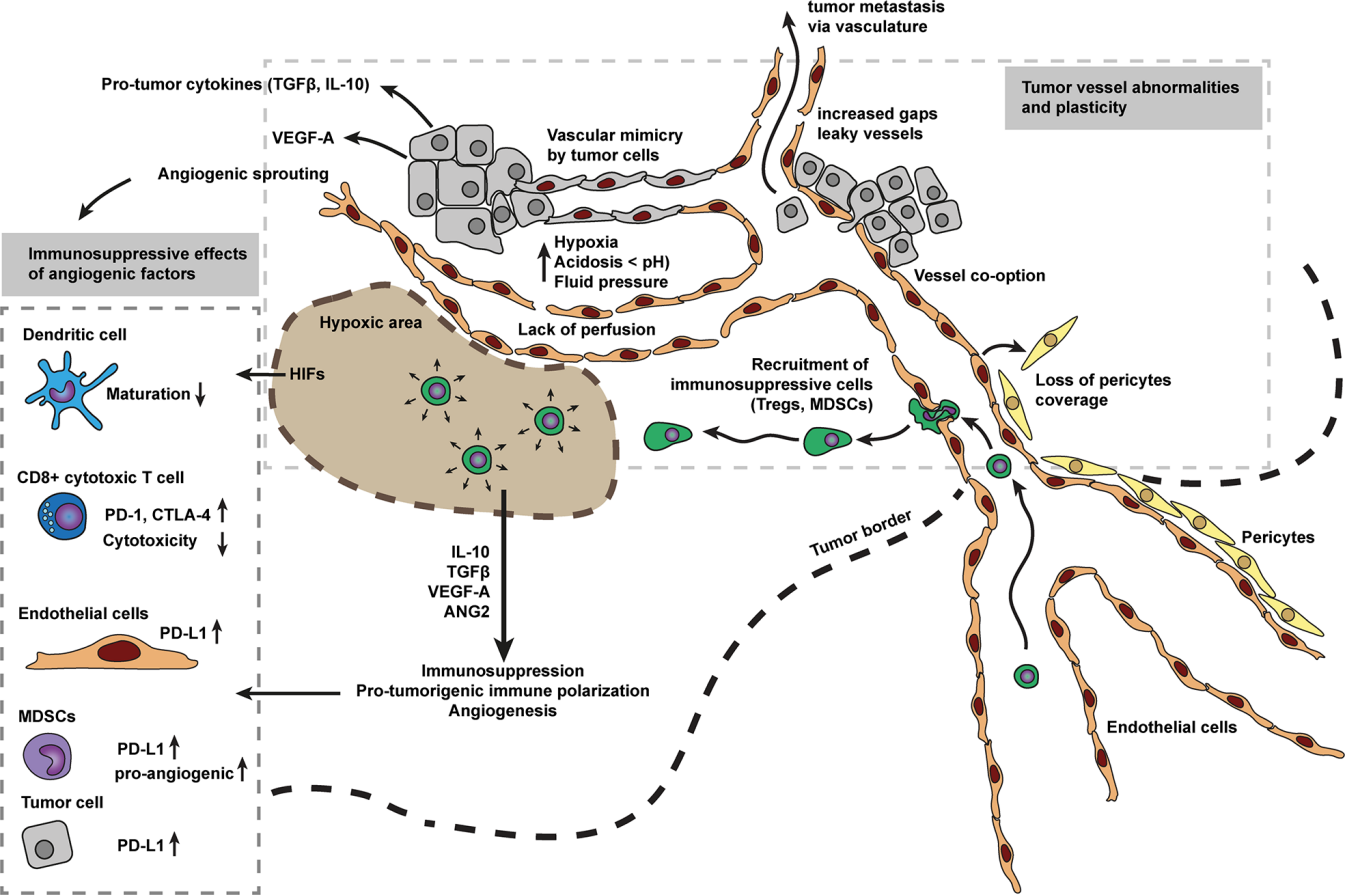
Tumor vessel abnormalities and angiogenesis as main driver for immunosuppression. The disorganized vascular network in tumors contains many intercellular gaps, abnormal sprouts, and holds significant areas which are not well perfused. The lack of vessel perfusion creates areas of hypoxic tumor tissue that in turn may reinforce angiogenesis, initiating a vicious cycle, as hypoxia is a key inducer of angiogenesis. Hypoxic zones in tumors also attract myeloid derived suppressor cells (MDSCs), tumor associated macrophages (TAMs) and regulatory T cells (Treg cells). Which in turn, respond to hypoxia by secreting various anti-inflammatory cytokines and growth factors (e.g., IL10, TGFβ, VEGF, and ANG2). These cytokines and growth factors have profound immunosuppressive effects on immune cells including pro-tumorigenic immune cell polarization, inhibition of cell maturation, inhibit cytotoxic capacity, increase expression of immune inhibitory checkpoints (e.g., PD-1, CTLA-4) and alter leukocyte recruitment. Tumor vessels are not well matured, characterized by low pericyte coverage. Low pericyte coverage causes improper and loose endothelial cell-cell contacts and allows tumor cells to metastasize *via* the bloodstream. Cancer endothelium is considered to be a heterogeneous population of cells, derived from vessel co-option, sprouting, bone-marrow, and/or vessel wall endothelial progenitor cells, transdifferentiated myeloid, and mesenchymal cells, or surprisingly derived from tumor cells mimicking endothelial cells.

In the 1980´s, it was proposed that tumors behave as a wounds that do not heal (44). There are many similarities between developing tumors and healing wounds, sharing overlapping biological players and pathways including recruitment of leukocytes and angiogenesis (45). Physiological angiogenesis in wounds typically occurs through vessel destabilization, sprouting, endothelial migration and proliferation, followed by resolution and stabilization of the new vessel. In cancer, angiogenesis is, however, characterized by a failure of the resolution phase. This results in a highly abnormal and disorganized vascular network that contains many intercellular gaps, abnormal spouts, and holds significant areas which are not well perfused (**Figure 1**). Intravital imaging in human melanoma tumors revealed that up to half of the neovasculature in tumors does not support blood flow (46). The lack of vessel perfusion creates areas of hypoxic tumor tissue that in turn may reinforce angiogenesis, initiating a vicious cycle, as hypoxia is a key inducer of angiogenesis (19). In cells, low oxygen levels are sensed by special proteins called hypoxia-inducible factors (HIFs) that in response to hypoxia translocate to the nucleus to initiate transcription of genes to promote cell survival in hypoxic conditions. These include glycolysis enzymes, which allow ATP synthesis in an oxygen-independent manner and pro-angiogenic proteins such as vascular endothelial growth factor (VEGF) and angiopoietin-2 (Ang2) (47).

In addition to induction of angiogenesis, pro-angiogenic proteins such as VEGF can also induce immunosuppressive functions (15) (**Figure 1**). One of the first described immunosuppressive mechanisms of VEGF was the inhibition of dendritic cell maturation with major consequences for presentation of tumor associated antigens (48). In addition, VEGF has also been implicated in inhibition of T cell function by inducing increased expression of immune inhibitory checkpoints including PD-1, CTLA-4, and Tim-3 mediating T cell exhaustion and anergy (9, 49). An additional factor found in the hypoxic tumor microenvironment are increased levels of HIFs (50). HIFs promote expression of ligands for immune inhibitory checkpoints (**Figure 1**). For instance, HIF1α increases the expression of PD-L1 on myeloid derived suppressor cells (MDSCs), tumor cells, DCs, and macrophages, hereby limiting T cell activation (20).

Hypoxic zones in tumors also attract MDSCs (21, 51, 52), tumor associated macrophages (TAMs) (22, 53) and regulatory T cells (Treg cells) (23, 54). In turn, these immune subtypes respond to hypoxia by secreting various anti-inflammatory cytokines and growth factors (e.g., IL10, TGFβ, VEGF and ANG2) in an attempt to relieve hypoxic stress (**Figure 1**). Many of these factors, however, contribute to immunosuppression and cancer escape through pro-tumorigenic immune cell polarization (24) or partial leukocyte trafficking blockade. Low hypoxia levels have also been reported to slow down tumor infiltrating lymphocytes (55). Collectively, this data shows how abnormal tumor vasculature contributes to immunosuppression in cancer.

## Consequences of Angiogenesis in Tumors for Leukocyte Trafficking

The vascular network provides a conduit for leukocytes to reach specific places in the body. The endothelium, paving the inner lining of the vascular network, acts as a gatekeeper for leukocytes, providing guidance where to exit the bloodstream. Leukocytes breaching the endothelium, also referred to as leukocyte transendothelial migration or diapedesis, is a common process that occurs during physiological as well as pathological conditions (56). For instance, immune cells travel in and out of lymphoid structures *via* high endothelial venules to perform immune surveillance or breach endothelial barriers to counter a bacterial infection. During acute inflammation, pro-inflammatory cytokines induce endothelial cell activation, creating local endothelial patches that express high levels of cell adhesion molecules (CAMs, i.e., Selectins, ICAM-1 and VCAM-1) at their surface to mark the exit site for leukocytes, close to the inflammatory site (57). This active endothelial state allows leukocytes to cross the endothelial barrier.

In general, every leukocyte diapedesis event occurs through the same fundamentally conserved multistep process, which was first described by Springer and Butcher et al, as a three step process, that became known as the multistep paradigm of leukocyte transendothelial migration (58, 59). The current steps include rolling, crawling, firm adhesion, arrest, docking structure formation, and transendothelial migration (56, 60).

Selectin-mediated interactions promote leukocyte recruitment to the site of inflammation or cancer progression (61). Endothelial-selectins, including E-selectin (also known as CD62-E, ELAM-1) and P-selectin (CD62-P) mediate the first step in the multistep process of leukocyte diapedesis (61). The primary selectin ligand on leukocytes, P-selectin glycoprotein ligand-1 (PSGL-1) triggers intracellular signaling through P-selectin or E-selectin binding mediating tethering and rolling of various hematopoietic cells (61, 62). Secondly, selective chemokines presented on the endothelial surface by heparan sulfate activate specific integrin subsets expressed by leukocytes (e.g., LFA-1 and VLA-4), and result in more stable leukocyte adhesion *via* CAM binding on the endothelium. Leukocyte binding to endothelial CAMs (e.g., ICAM-1 and VCAM-1) support the adhesion phase including crawling, firm adhesion, arrest and docking structure formation (63, 64). The last step, transmigration, occurs either between two or more adjacent endothelial cells (paracellular route) or through the cell body of an individual endothelial cell (transcellular route). In both cases, an F-actin ring within the endothelium prevents vascular rupture and leakage while leukocytes pass the endothelial cell layer (57).

In addition, several processes shape the multistep paradigm and may refine leukocyte trafficking at predefined endothelial “hotspots” (60). These include the recruitment of leukocytes towards an optimal concentration of chemokines (chemotaxis), the density of adhesion molecules (haptotaxis), cellular stiffness (durotaxis) and migrating of leukocytes along the path of least resistance (tenertaxis), which also governs decisions making where to exit the endothelium (65, 66).

To infiltrate a tumor and to become part of the TME, immune cells must adhere and breach the endothelial lining of cancer vessels. The vasculature in cancer is, however, not normal. On a genetic level tumor endothelium differs from those located in normal resting tissues. Cytogenetic studies on epithelial renal cell carcinoma and nonepithelial (liposarcoma and melanoma) tumors have reported chromosomal aberrations and aneuploidy in tumor endothelial cells (67, 68), indicating that tumor endothelial cells, like tumor cells, can be genetically instable (67). On the transcription level, two particularly interesting studies compared the gene expression profiles of endothelial cells isolated from five distinct tumors and eight resting normal tissues (69, 70). Of the transcripts identified, 25 were overexpressed in tumor versus normal endothelium. Most of the genes identified had expected roles in angiogenesis and cell-cycle control, but those specific to tumor endothelium were mainly cell surface molecules of unknown function. Another intriguing study revealed that the most downregulated gene classes in tumor endothelial cells involved antigen presentation (i.e., Major histocompatibility complex class I, II), immune cell homing molecules (i.e., ICAM-1), and chemotaxis related molecules (i.e., CCL2, CCL18, and IL6), which might explain how tumor cells gain immuno-tolerance (71). Furthermore, single-RNA sequencing revealed the breadth in heterogeneity of lung tumor endothelial cell phenotypes in human patients and mouse tumor model systems. Tip tumor endothelial cells were congruent across species and models and shared conserved markers (72).

On a cellular level the origin of cancer endothelium considers various cellular sources (**Figure 1**). Endothelial patches are homogeneous, and can be derived from angiogenesis-induced spouts, co-opt nearby vessels, or are derived from bone-marrow or vessel wall endothelial progenitor cells (73, 74). Additionally, vessels may also be derived from unexpected sources, including endothelial cells derived from transdifferentiated myeloid and mesenchymal cells, or even from tumor cells mimicking endothelial cells (74). The multi-source origin of endothelial cells in abnormal tumor vessels may explain the plasticity of tumor vessels to escape anti-angiogenesis therapy (74).

Gene expression profiles in cancer endothelial cells might also depend on tumor size. For instance, it was suggested that smaller tumors express different angiogenic gene profiles compared to larger tumors. One study that explored this relationship found that genes associated with angiogenesis did not change during the different growth stages. However, cell surface molecules involved in leukocyte trafficking, i.e., ICAM-1 and VCAM-1 were downregulated in intermediate and large tumors (75). Moreover, several papers have shown a clear link between angiogenesis-modulatory molecules such as VEGF and basic fibroblast growth factor (bFGF) and downregulation of CAMs on tumor endothelial cells (76, 77). Together this data shows how angiogenesis modulating factors interfere with CAM expression on tumor endothelium which alters leukocyte infiltration into tumors.

## Cancer Endothelium a Selective Barrier for Leukocytes

As evidenced by the various immune phenotypes in cancer, i.e., desert, immune-excluded and inflamed tumors the severity of impaired leukocyte entry into tumors ranges from a partial blockade to a complete immune infiltration blockade into tumors. The fact that some immunosuppressive immune cells such as regulatory T cells (Tregs) and MDSCs can enter the TME while cytotoxic effector cells are excluded also suggests a selective barrier at the level of the endothelium. What factors contribute to this selectivity is currently debated and of ongoing investigation.

**Figure 2:**
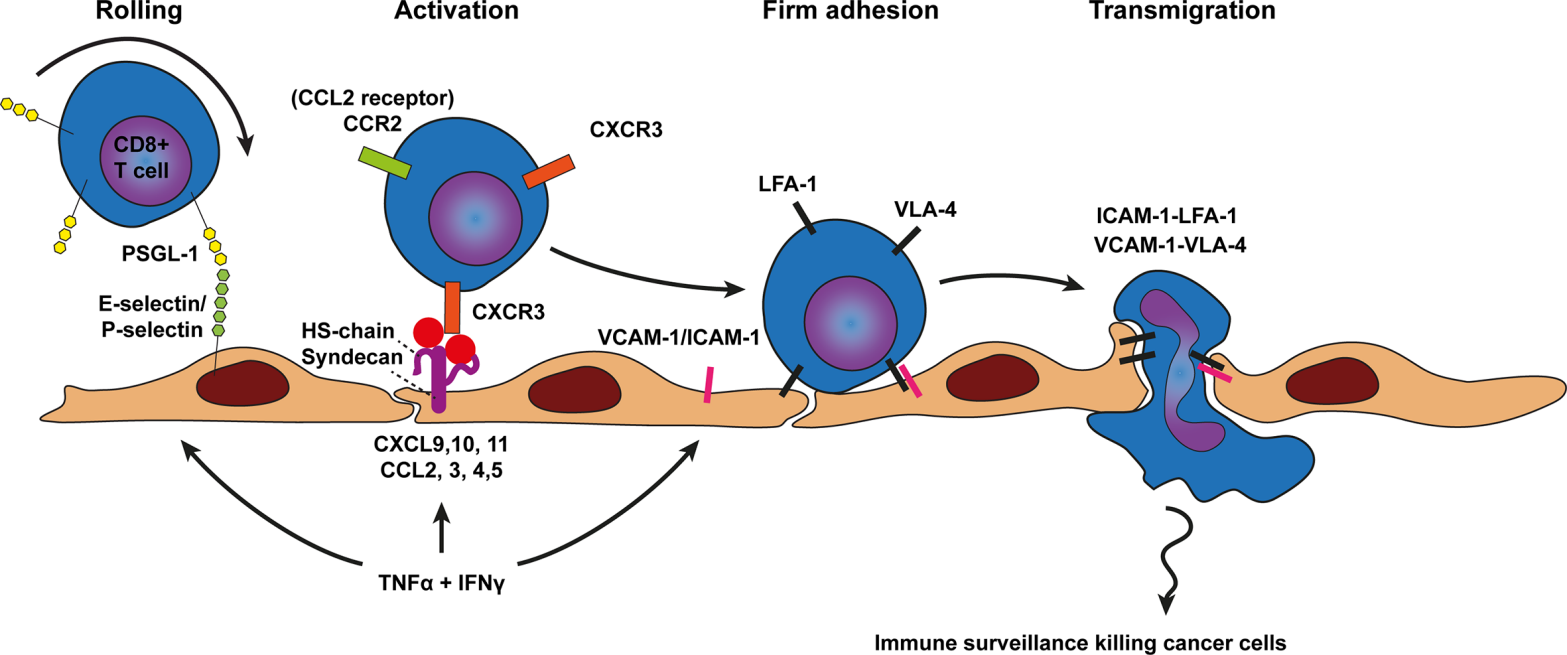
Model of CD8+ T cell trafficking across cancer endothelium. Transmigration of CD8+ T cells across cancer endothelium is initiated by a tethering and rolling behavior which dependents on E-selectin and P-selectin binding to most likely P-selectin glycoprotein ligand 1 (PSGL1) on CD8+ T cells. CD8+ T cell activation, shown in the next step, occurs predominantly through the CD8+ T cell attracting chemokines CCL2, CXCL9, 10, and 11 which are presented on the endothelium by heparan sulfate (HS) to the chemokine receptors on CD8+ T cells (e.g., CCR2 and CXCR3). Heparan sulfate is depicted here as the heparan sulfate proteoglycan (HSPG) syndecan but other HSPGs might also be involved. Chemokine presentation activates CD8+ T cell integrins (i.e., LFA-1 and VLA-4) and results in more stable CD8+ T cell adhesion *via* LFA-1-ICAM-1 and VLA-4-VCAM-1 binding. Firm adhesion is followed by transendothelial migration which also involves LFA-1-ICAM-1 and VLA-4-VCAM-1 interactions. TNF-α and INFγ derived from various sources in the tumor microenvironment result in concomitant upregulation of cell adhesion molecules as well as presentation of CD8+ T cell specific chemokines by HS on the endothelium.

**Figure 3:**
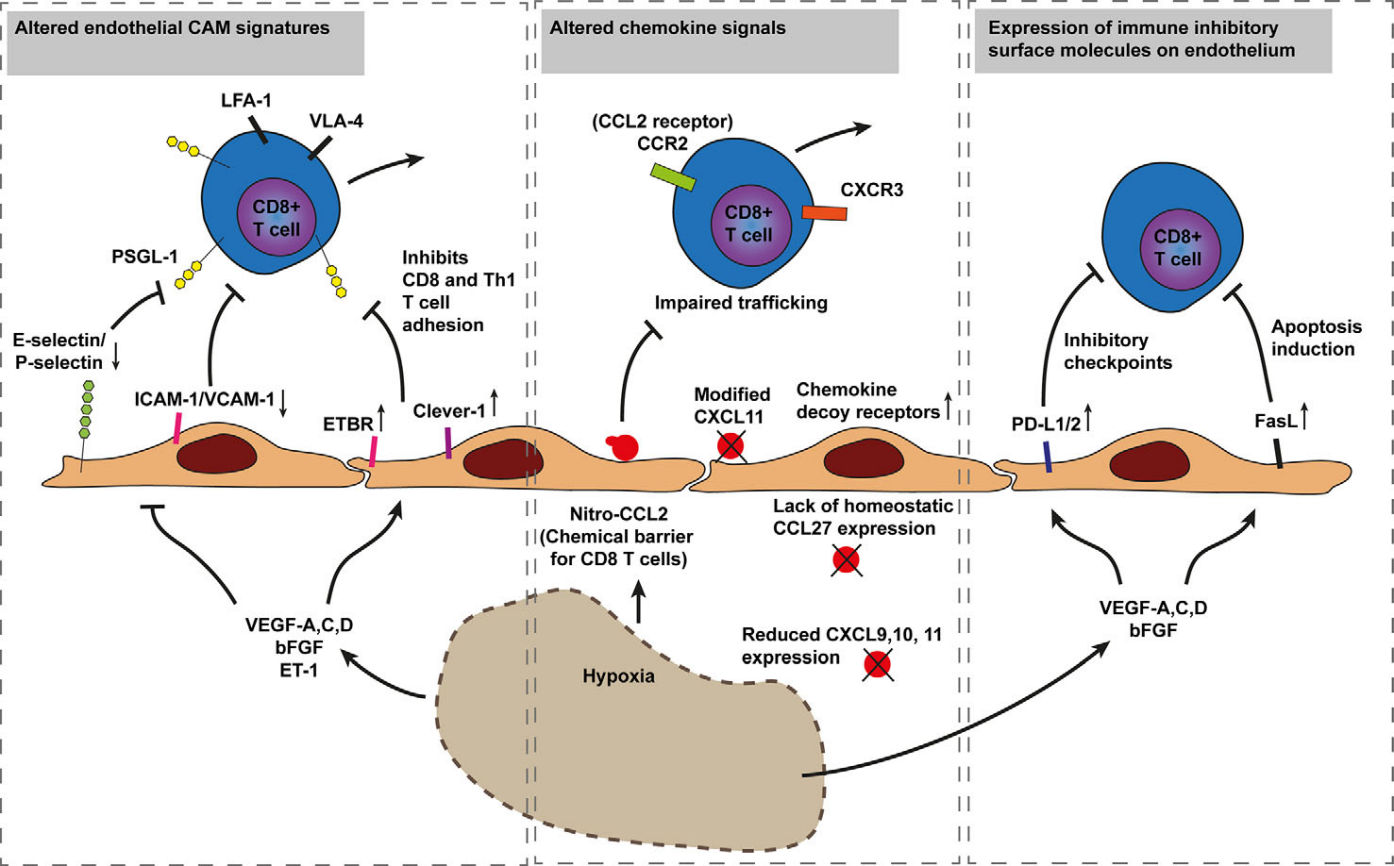
Model of cancer immune evasion through inhibition of cytotoxic T cell recruitment. Hypoxia driven angiogenesis impairs CD8+ T cell trafficking in three established ways. The first mechanism involves inhibition of T cell adhesion through downregulation of endothelial E-selectin/P-selectin, ICAM-1, and VCAM-1 or increased expression of T cell repelling molecules such as ETBR and Clever-1 by various hypoxia induced factors (i.e., VEGF-A,C,D, bFGF, and ET-1). The Second involves altered chemokine expression and chemical barriers. For instance, nitrosylation of CCL2 by reactive nitrogen species blocks CD8+ T cell recruitment while improving recruitment of MDSCs. Additionally, a lack of CD8+ T cell specific chemokines or the increased expression of chemokine decoy receptors, scavenging away CD8+ T cell recruiting chemokines, impair CD8+ T cell tumor infiltration. The third mechanism occurs through immunosuppressive barriers as increased hypoxia and angiogenesis modulatory factors increase endothelial PD-L1/2 and FasL expression inducing T cell exhaustion and apoptosis, respectively. Endothelin B receptor (ETBR), common lymphatic endothelial, and vascular endothelial receptor-1 (Clever-1, also known as stabilin-1). Programmed death-ligand 1 (PD-L1), Fas ligand (FasL), P-selectin glycoprotein ligand 1 (PSGL1).

**Figure 4:**
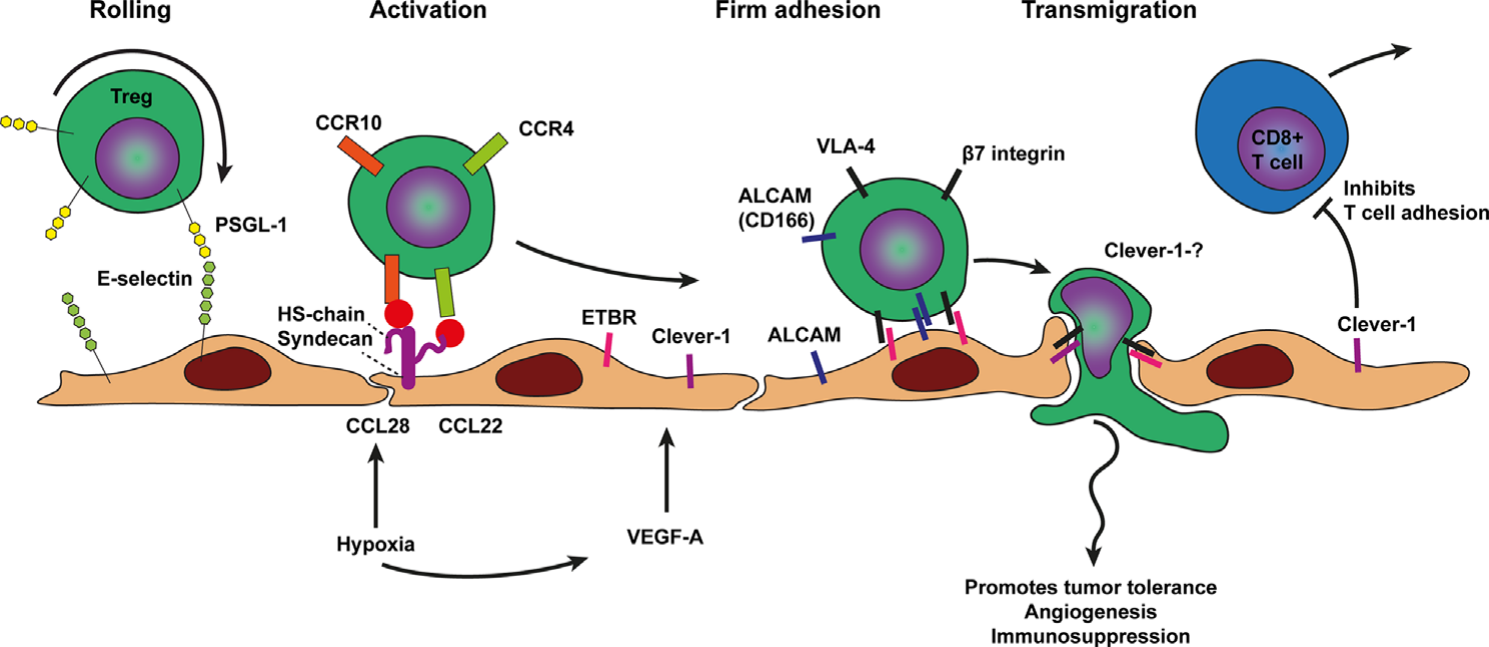
Model of T regulatory cell trafficking across cancer endothelium. The selective trafficking of Treg cells across cancer endothelium is initiated by a tethering and rolling behavior which dependents on E-selectin binding to most likely P-selectin glycoprotein ligand 1 (PSGL1) on Treg cells. Treg cell activation, shown in the next step, involves the hypoxia induced chemokines CCL28 and CCL22 which are presented on the endothelium by heparan sulfate (HS) to the chemokine receptors on Treg cells, i.e., CCR10 and CCR4 respectively. Heparan sulfate is depicted here as the heparan sulfate proteoglycan (HSPG) syndecan but other HSPGs might also be involved. Chemokine receptor engagement activates Treg cell integrins most likely VLA-4 and β7 integrin which results in more stable Treg cell adhesion *via* Clever-1, ALCAM, and ETBR binding. The exact ligands for ALCAM, Clever-1, and ETBR are not well described. Firm adhesion is followed by transendothelial migration which likely also involves Clever-1 and ETBR. VEGF-A derived from various sources in the tumor microenvironment and hypoxia may result in concomitant upregulation of cell adhesion molecules as well as presentation of Treg cell specific chemokines by HS on the endothelium. Tumor infiltrated Treg cells promote tumor tolerance, angiogenesis, and immunosuppression.

**Figure 5:**
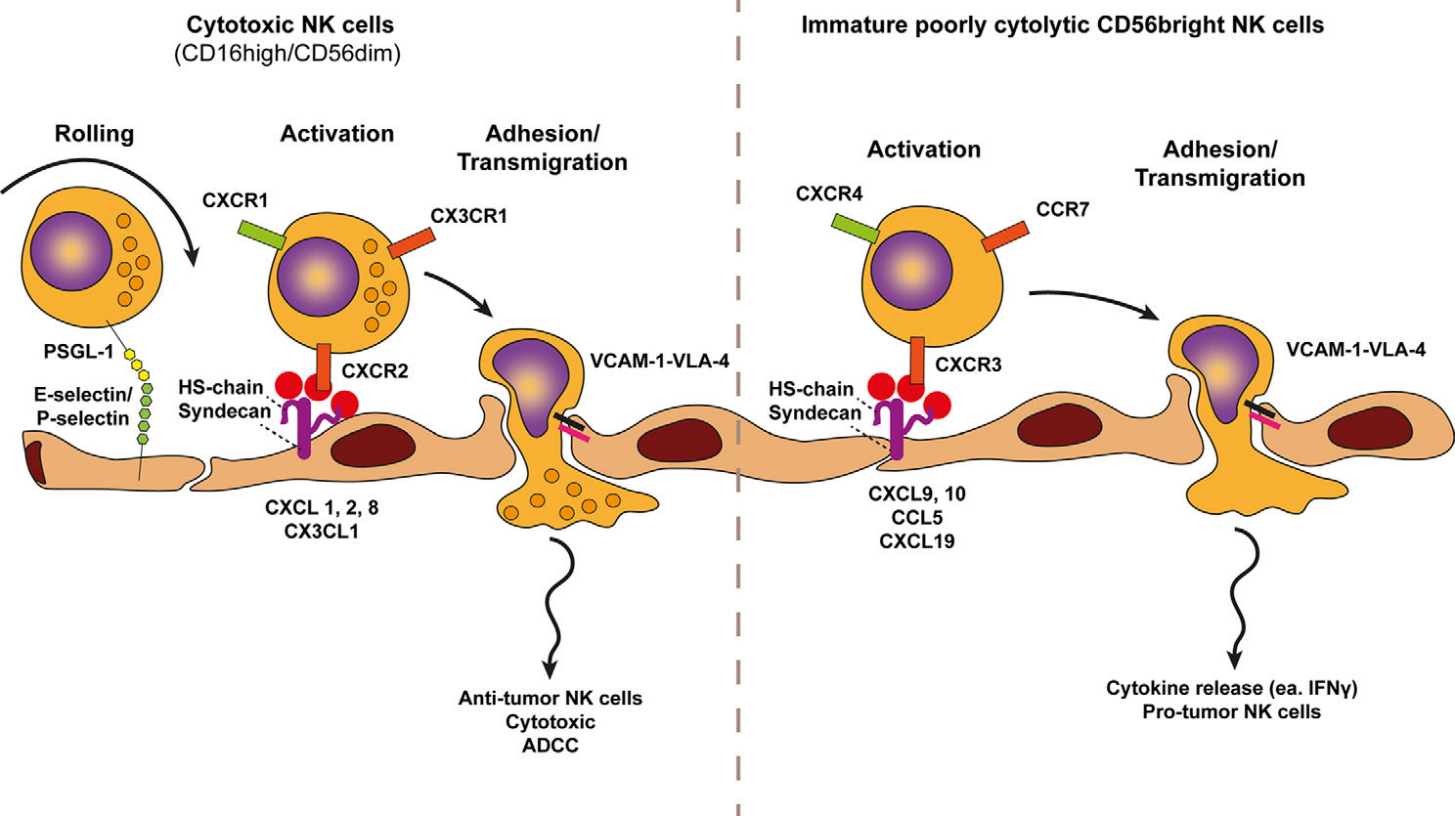
Model of NK cell trafficking across cancer endothelium. A conventional view on NK cells subsets defines two NK cell types, the cytotoxic CD56dimCD16pos and the immature cytokine secreting CD56brightCD16low/neg NK cell variants. The selective trafficking of both NK cell subsets across cancer endothelium are proposed to involve E-selectin and P-selectin binding to most likely P-selectin glycoprotein ligand 1 (PSGL1) on NK cells, initiating a tethering and rolling behavior. Subsequent activation of each NK cell type occurs through subtype specific chemokines presented by heparan sulfate (HS) on the endothelium. Heparan sulfate is depicted here as the heparan sulfate proteoglycan (HSPG) syndecan but other HSPGs might also be involved. The chemokine receptors of cytotoxic (anti-tumor) NK cells, i.e., CXCR2, CXCR1, and CX3CR1 are activated by CXCL1,2,8 and CX3CL1 gradients whereas the chemokine receptors of immature cytokine producing NK cells (pro-tumor), i.e., CXCR4, CCR7, and CXCR3 are activated by increased presentation of CXCL9/10, CXCL19/21, and CCL5 gradients. Chemokine presentation activates NK cell integrins (i.e., VLA-4) and results in more stable NK cell adhesion *via* VLA-4-VCAM-1 binding. Firm adhesion is followed by transendothelial migration which also involves VLA-4-VCAM-1 interactions.

**Figure 6:**
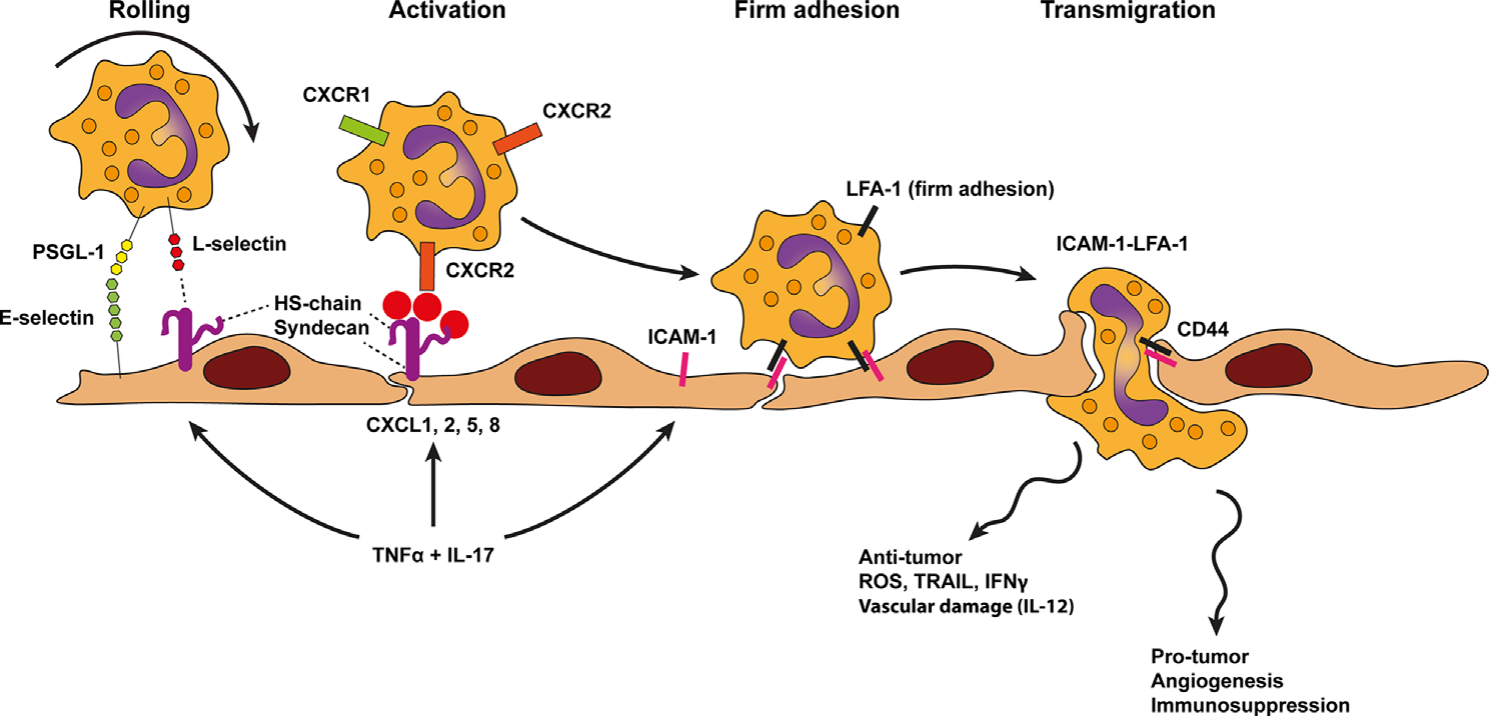
Model of neutrophil trafficking across cancer endothelium. Neutrophil diapedesis in the context of cancer likely relies on E-selectin and ligands, such as P-selectin glycoprotein ligand 1 (PSGL1), on neutrophils which initiate rolling on the endothelium. Rolling is further stabilized by lymphocyte (L)-selectin binding to endothelial-cell heparan sulfate (HS). Heparan sulfate also presents neutrophil specific chemokines such as CXCL1, 2, 5, 8 to chemokine receptors on neutrophils (i.e., CXCR1 and CXCR2). This process activates neutrophil integrins and results in more stable leukocyte adhesion *via* LFA-1-ICAM-1 binding, followed by transendothelial migration using the same molecules. TNF-α and IL-17 derived from various sources in the tumor microenvironment result in concomitant upregulation of cell adhesion molecules as well as presentation of neutrophil specific chemokines by HS on the endothelium. In contrast to inflammation neutrophil trafficking across cancer endothelium might be Mac-1 independent. Moreover, an additional role for homotypic CD44 interactions have been proposed to guide transmigration.

**Figure 7:**
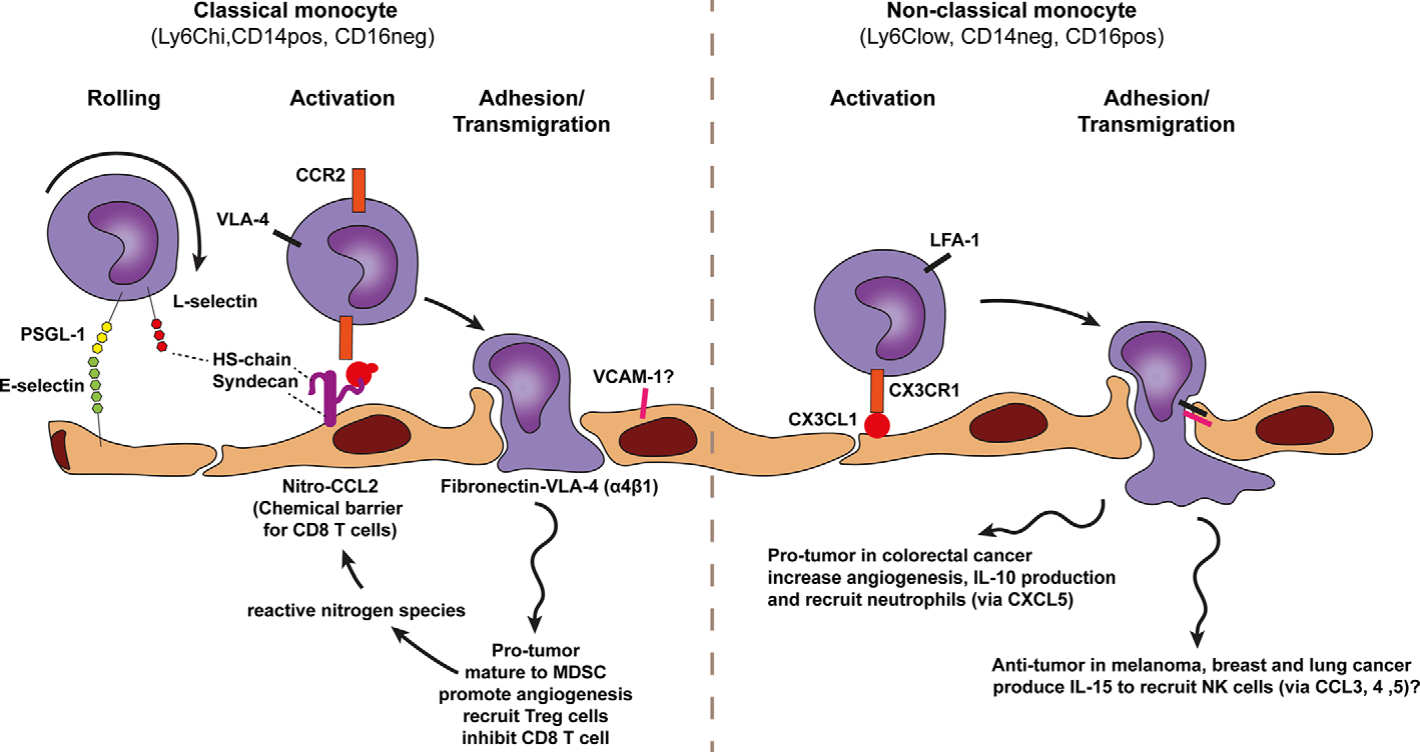
Model of monocyte trafficking across cancer endothelium. A conventional view on monocyte subsets defines two monocyte types, the classical monocyte (Ly6Chi CD14pos CD16neg and the non-classical monocyte Ly6Clow CD14neg CD16pos. Both subsets show anti-tumor as well as pro-tumor effects which are context-dependent. The selective trafficking of both monocyte types are proposed to involve E-selectin binding to most likely P-selectin glycoprotein ligand 1 (PSGL1) on monocytes, initiating a tethering and rolling behavior, which under some conditions also involves lymphocyte (L)-selectin. L-selectin may stabilize rolling by binding to endothelial-cell heparan sulfate (HS). Subsequent activation of each monocyte type occurs through subtype specific chemokines presented by heparan sulfate (HS) on the endothelium. Heparan sulfate is depicted here as the heparan sulfate proteoglycan (HSPG) syndecan but other HSPGs might also be involved. The CCR2 chemokine receptor on classical monocytes is proposed to be activated by nitrosylation modified-CCL2 (activation can also performed by other additional chemokines) whereas non-classical monocytes are proposed to be activated in a CX3CR1-CX3CL1 dependent manner. Chemokine receptor engagement activates monocyte integrins. For classical monocytes this is most likely VLA-4 and for non-classical monocytes LFA-1 has been proposed which results in more stable monocyte adhesion *via* VCAM-1 and ICAM-1 binding, respectively. Classical monocytes might also bind exposed fibronectin directly *via* VLA-4. Classical monocytes are able to polarize to MDSCs and promote angiogenesis and production of reactive nitrogen species.

Selective immune cell trafficking is regulated by a unique combination of endothelial specific CAMs and surface bound chemokines that interact with matching counterparts (integrins and chemokine receptors) expressed by specific leukocyte subtypes. In the context of cancer there are multiple pathways and mechanisms described that drive selective immune cell trafficking. It is important to realize that some of these pathways exist only temporally as they are continuously subjected to changing microenvironments as tumor development progresses and pathways might differ across tumor and tissue type. For many tumor types there is limited information on this topic so the represented information is based on a selected number of studies and may therefore not cover all tumor microenvironments and tumor types. Models (**Figures 2**–**7**) are simplified to improve understandability and serve as a starting point to understand and unravel the complete breadth of molecules involved in selective immune cell trafficking of diverse immune subtypes across diverse tumor types and tissues. While many parallels between inflammation and cancer development have been described, it remains to be evaluated how the various selectins, CAMs and chemokines mediate selective trafficking in cancer. We will now discuss the most established mechanisms of selective immune cell trafficking in cancer.

### CD8 T Cell Trafficking Across Cancer Endothelium

In non-lymphoid tissues, resting endothelial cells lining small capillaries are usually refractory to leukocyte adhesion. Tissue entry *via* the vasculature occurs only occasionally by monocytes and a few recirculating T lymphocytes. L-selectin controls the capacity for naïve T cells to migrate to the lymph nodes, whereas P- and E-selectin capture activated T cells on activated inflamed endothelium to initiate their migration into non-lymphoid tissues (62). In tumors, initiation of T cell infiltration has been suggested to start with an initial infiltration of a few T cells, followed by a large influx of both specific and nonspecific T cells (78).

To enter tumor tissue *via* the vasculature CD8+ T cells first establish weak interactions through selectin based adhesions, likely mediated by P and E-selectin binding to PSGL-1 on the T cell (62, 79). These weak interactions slow down the leukocyte, inducing a slow-rolling behavior (**Figure 2**). Slow-rolling leukocytes subsequently interact with surface bound chemokines on the endothelium using a specific repertoire of chemokine receptors expressed by the cytotoxic T cell. A major chemokine receptor expressed by activated tumor infiltrating leukocytes (TILs) is CXCR3, which has been found on many CD8+ T cells that infiltrated breast, colon and melanoma tumors (31, 80–84). CXCR3 recognizes CXCL9 (Mig) and CXCL10 (IP10) which is presented on the inner lining of cancer vasculature. CXCR3 binding to CXCL9 and CXCL10 activate integrins (e.g., VLA-4) expressed on the cell surface of CD8+ T cells. Activated VLA-4 (also known as α4β1) enables transient interactions with VCAM-1 expressed on the cell surface of cancer endothelium supporting adhesion and transmigration (**Figure 2**).

Solid tumors expressing CXCL9 and CXCL10 recruited more CD8 T cells and showed better response to immunotherapy (85, 86). There are also tumors in which CXCL9 and 10 are low, which may explain why some tumors have low levels of CD8 T cell infiltration (80, 82). In addition to the C-X-C motif containing chemokines, T cells are predominantly recruited in some tumors *via* chemokines C-C motif ligands including CCL2, CCL3, CCL4, and CCL5 (80). Of these chemokines, CCL2 has been shown to increase homing of chimeric antigen receptor T cells (CAR T cells) that express functional CCR2 (receptor for CCL-2) (87).

Furthermore, cytokines may also modulate CD8 T cell trafficking as cytokines released by immune or cancer cells have shown to affect the expression of selective chemokines and CAMs at the surface of cancer endothelium. For instance, TNFα is known to increase endothelial adhesion molecules, in particular E-selectin, ICAM-1 and VCAM-1 (88). However, TNF alone has limited influence to enhance T cell entry into tumors this requires the presence of additional cytokines/chemokines. For instance interferon gamma (IFNγ) a key effector molecule released by activated cytotoxic T lymphocytes. Interestingly, the combination of TNF and IFNγ amplifies endothelial ICAM-1 and VCAM-1 surface expression and simultaneously provides selectively to CD8+ T cell recruitment by inducing several chemokines specific for CD8+ T cells including CXCL9/10/11 and CCL5 (89–91). Several studies have examined which endothelial adhesion molecules enable infiltration of effector CD8+ T-cells into tumors, either directly using genetic knockout models (and blocking antibodies) or as a correlation (10, 30, 31, 92–97). Increased ICAM-1 and VCAM-1 expression by the tumor vasculature in response to the pro-inflammatory agent CpG-ODN correlated with improved CD8+ T cell trafficking in a mouse model of pancreatic islet cell carcinoma (RIP1-Tag5) (94). In addition, in glioblastoma increased infiltration of CD8+ T cells also correlated with the expression of ICAM-1 and VCAM-1 on the vessel surface (95) Either, ICAM-1 and VCAM-1 blocking antibodies reduced T cell infiltration. Combined ICAM-1/VCAM-1 blockade showed the strongest impairment of T cell infiltration in glioblastoma (95). Furthermore, analyses of partly rejected B16/BL6 melanoma tumors revealed increased expression of ICAM and VCAM by the tumor vasculature, providing support for the idea that expression of these molecules strongly correlates with immune cell infiltration and tumor rejection (30). LFA-1 deficient mice failed to reject immunogenic MC57 fibrosarcoma tumors showing an important role for LFA-1 in CD8+ T cell recruitment and tumor rejection (96). Moreover, in a model for ovarian carcinoma and fibrosarcoma VLA-4-VCAM-1 as well as LFA-1-ICAM-1 interactions have been shown to mediate T cell infiltration in response to IL-12 treatment (92). In a separate ovarian cancer study anti-ICAM-1 blocking antibodies only partly blocked CD8+ T cell infiltration, suggesting that additional molecules like VCAM-1 may mediate CD8+ T cell extravasation as well (10). In other studies, the infiltration of CD8+ T cells was shown to be mediated by either ICAM-1 or VCAM-1 alone. In a study that combined vaccination with VEGF receptor tyrosine kinase (RTK) inhibitor, in a M05 mouse breast cancer model, it was shown that VCAM-1 blocking antibodies or blocking the CXCR3 receptor impaired CD8+ T cell infiltration and therapeutic efficacy (31). Evaluating ICAM-1 expression in a pancreatic islet cell carcinoma (RIP1-Tag5 model), a colorectal cancer model (CT26) and B16-OVA tumors showed that extravasation of CD8+ T cells was E-selectin, P-selectin and ICAM-1 dependent, which could be blocked using corresponding blocking antibodies (93). The lack of CD8 T-cell infiltration into subcutaneous B16-OVA melanoma was confirmed in ICAM-1−/− mice (93), although conflicting it was shown that T cell penetration into B16 melanoma did not differ between wild-type and ICAM-1-/- mice (98). Moreover, also in models of pancreatic and hepatocellular cancer no significant differences in CD8+ T cell adhesion and infiltration were found in ICAM-1 and MAC-1 deficient mice. Instead an important role for LFA-1 was found (97). Perhaps in pancreatic and hepatocellular cancer LFA-1 mediated CD8+ T cell extravasation *via* its other ligands including ICAM-2, ICAM-3, ICAM-4, ICAM-5, and JAM-A (99), which might play an important role to establish firm adhesion to cancer endothelium Together this supports a model for CD8+ T cell transmigration across cancer endothelium that occurs predominantly through the CD8+ T cell attracting chemokines CCL2, CXCL9, and CXCL10, as well as *via* CD8+ T cell integrins, LFA-1 and VLA-4, and endothelial E-selectin, ICAM-1 and VCAM-1. The direct role of VCAM-1, ICAM-1, and ligands for CXCR3 or other chemokine receptors in mediating T-cell entry into additional murine and human tumors and the concomitant effects on other immune subtypes still needs to be thoroughly examined.

### Cancer Immune Evasion Through Inhibition of Cytotoxic T Cell Recruitment

The direct effects of hypoxia, abnormal vasculature and hypoxia induced angiogenic factors such as VEGF-A, C, D, bFGF, EGFL7 have profound immunosuppressive effects and block T cell infiltration in tumors in various ways (**Figure 3**). First, angiogenic factors reduce endothelial VCAM-1 expression which limits T cell infiltration (76, 77).

Second, T cell trafficking to cancer is blocked through several mechanisms that interfere with chemokine signaling. For instance, T cell infiltration has been shown to be blocked by chemokine decoy receptor expression on tumor vessels (like DARC) that scavenge the CD8+ T cell recruiting chemokines CCL2 and CCL5 (100). Chemokine decoy receptors can also increase trafficking of immunosuppressive cells, for instance through downregulation of chemokine decoy receptor D6 that normally keeps CCL22 expression in check, increasing Treg cell recruitment (101). Moreover, CD8+ T cell infiltration can also be impaired through VEGF-A-mediated reduction of CD8+ T-cell recruiting chemokines CXCL9, 10, and 11. Reduced chemokine presentation can occur through downregulation or proteolytic processing as has been shown for CXCL11, which is an important chemokine recruiting CXCR3 expressing effector T cells. Dysfunctional CXCL11 impairs binding and signaling of the chemokine, ultimately reducing lymphocyte infiltration in tumors (102). Furthermore, loss of homeostatic chemokine expression can interfere with T cell infiltration as well. In skin tumors, loss of CCL27, a chemokine constitutively expressed by normal keratinocytes, impaired T-cell homing to cutaneous tumors accelerating tumor outgrowth in a tumor mouse model of B16F10 melanoma (12).

In addition, interference with T cell attracting chemokines has also been described to involve nitrosylation. In response to reactive nitrogen species (RNS) tumor cells modify CCL2 intro nitrosylated CCL2. Nitro-CCL2 recruits monocyte derived suppressor cells and repels trafficking of cytotoxic T cells and Th1 effector cells, confining T cells in stromal regions that surround cancers cells (13).

Another mechanism of endothelial anergy (lack of responsiveness) involves the binding of endothelin-1 to endothelin B receptor (ETBR), which prevents T cell adhesion to cancer endothelium, even in the presence of the inflammatory cytokine TNFα (10, 103). In oesophageal squamous cell carcinoma high ETBR expression correlated with angiogenesis and lower survival rates (104).

Finally, cancer endothelial cells are able to increase FasL expression in response to VEGF-A. Upregulation of FAS ligands on cancer endothelium has been suggested to induce T cell apoptosis (11). In addition to Fas ligands, cancer endothelium can also express increased levels of the immune inhibitory checkpoints PD-L1 and PD-L2 (26). The expression of PD-L1/2 may initiate T cell anergy and exhaustion even before entering the tumor microenvironment.

### T Regulatory Cell Trafficking Across Cancer Endothelium

As outlined previously, tumor growth results in hypoxia, which inducesangiogenesis in tumors. Because angiogenic tumor vessels are often dysfunctional and not well perfused angiogenesis in tumors is likely to result in more hypoxia. These hypoxic conditions in turn promote the recruitment of T regulatory cells (Treg cells). which promotes tolerance and angiogenesis to support tumor growth and dissemination (23). L-selectin expression is required for proper trafficking and distribution of Tregs cells under physiological conditions (105). In an inflammatory model Tregs have been shown to perform rolling under high shear stress using tether and sling formation and the authors predicted that Treg cells will also show P- and E-selectin-dependent homing to sites of inflammation (106). The precise involvement of selectin based Treg cell rolling in cancer recruitment requires further investigation. Treg cells express CCR4 and are recruited into the tumor micro environment in response to CCL22, which is produced mainly by macrophages and tumor cells (25) (**Figure 4**). Another chemokine shown to recruit Treg cells is the hypoxia related chemokine CCL28 (23) which may reinforce the vicious cycle of hypoxia induced angiogenesis resulting in more abnormal vasculature and hypoxia. In addition, Tregs exposed to hypoxia show increased CXCR4 surface expression which in turn can interact with its ligand stromal-derived-factor-1 (SDF-1 also called CXCL12) to mediate infiltration of Treg cells into breast cancer (107).

Several angiogenic factors including VEGF-A,C,D, bFGF, and Egfl7, increased expression of the ETBR (10, 103). Glioblastomas with higher numbers of ETBR-expressing vessels showed lower infiltration by cytotoxic T cells and higher numbers of regulatory T cells. Cytotoxic T cells infiltrated around ETBR-negative blood vessels, but were absent around vessels expressing ETBR (28). The common lymphatic endothelial and vascular endothelial receptor-1 (Clever-1, also known as stabilin-1) also selectively mediates transmigration of Tregs across cancer endothelium. In tumors, VEGF-A induces upregulation of Clever-1, recruiting Tregs while repelling CD8 T cell binding (27). Clever-1 knockout mice show impaired Treg trafficking while trafficking of CD8 T cells was unchanged (108). Clever-1 knockout mice also had impaired tumor migration *via* lymphatics (108).

In addition to ETBR and Clever-1, several adhesion molecules have been described to regulate selective transmigration of Tregs across cancer endothelium and might be cancer type specific. In pancreatic carcinoma, selective transmigration of Tregs involved the mucosal adressin cell adhesion molecule-1 (MAdCAM-1), VCAM-1, E-selectin, and activated leukocyte cell adhesion molecule (ALCAM, also known as CD166). Blocking antibodies against the ligands β7 integrin, L-selectin, and CD166 specifically expressed on the surface of Treg cells impaired Treg trafficking (29). In contrast, blocking antibodies against P-selectin, ICAM-1 and ICAM-2 did not block Treg transmigration (29).

### NK Cell Trafficking Across Cancer Endothelium

NK cells play an active role in immune surveillance which is particularly effective for hematological tumors, early stage solid tumors and metastatic cells circulating in the blood (109, 110). Conversely, NK cells are less effective in controlling advanced solid tumors that are characterized by an abundant variety of immunosuppressive factors typical for a tumor microenvironment. These include the direct effects of hypoxia and angiogenesis factors on cancer and immune cells increasing the amount of surface expressed immune inhibitory checkpoints, such as PD-L1 and B7-H3 (9, 111) as well as immunosuppressive cytokines such as TGFβ released by tumor cells and tumor associated macrophages (112–114).

Another aspect that impacts the performance of NK cell mediated immune surveillance is the low frequency and/or type of NK cell recruited to the tumor. The two major functions of NK cells, i.e., cytokine production and cytotoxic activity are conventionally associated with distinct NK cell subsets. Whereas cytotoxic activity is mostly confined to NK cell variants identified as CD56dimCD16pos, production of cytokines, i.e., IFNγ occurs mainly by immature CD56brightCD16low/neg NK cells (115). In line with tumors escaping immune surveillance, cytotoxic CD56dimCD16pos NK cells are sporadically found in advanced solid tumors whereas immature CD56brightCD16low/neg NK cell variants represent the majority of tumor infiltrated NK cells.

The adhesion of NK cell variants to cancer endothelium shows similarities to that of CD8 T cells, starting with selectin-induced rolling, followed by VCAM-1-VLA-4 mediated adhesion and transmigration (**Figure 5**). Mice deficient in E- and P-selectin were defective in NK cell recruitment to subcutaneous Lewis lung carcinoma (LLC) and MC38 tumors, showing the relevance of these molecules in NK cell tumor infiltration (116). IL-2-induced NK cell infiltration into lung, liver and subcutaneous B16 melanoma was mediated by endothelial VCAM-1 interacting with VLA-4 on NK cells. NK cell infiltration was neither blocked by anti-ICAM-1 nor LFA-1 blocking antibodies (117). Selective recruitment however, is determined by distinct chemokines presented at the surface of cancer endothelium. Immature pro-tumor CD56brightCD16low/neg NK cell recruitment occurs *via* increased expression of CXCL9, CXCL10, CXCL19, CXCL21, and CCL5, and reduced expression of CXCL2, CX3CL1, CXCL1, and CXCL8 (118). Conversely, CXCL2, CX3CL1, CXCL1, and CXCL8 are important recruiters of cytotoxic CD56dimCD16pos NK cells into the TME. Of note, CXCL9 and CXCL10 recruiting pro-tumor immature CD56brightCD16low/neg NK cells are also involved in the recruitment of cytotoxic CD8 T cells.

### Neutrophil Trafficking Across Cancer Endothelium

In a physiological setting, neutrophils play a vital role to counter microbial infections and have supportive functions during wound healing. In cancer, however, neutrophils have shown to support tumor growth and progression through angiogenesis induction and immunosuppressive functions (119, 120). In general, neutrophil transmigration across cancer endothelium resembles that of inflammation. Heparan sulfate deficiency in mice showed reduced L-selectin and chemokine-mediated neutrophil trafficking in an inflammatory model, showing two important functions of endothelial heparan sulfate: acting as a ligand for L-selectin and presenting chemokines at the luminal surface of the endothelium (63, 64). Furthermore, the use of L-selectin and E-selectin deficient mice in lung and CRC models have shown that selectin-mediated recruitment of neutrophils support cancer metastasis (121, 122). In inflammatory settings the integrin LFA-1 (CD11a/CD18, αLβ2) is required to establish firm adhesion and transmigration of neutrophils across vascular beds. Mice lacking CD18 (β2 integrin) have impaired infiltration of neutrophils in solid tumors, indicating that this integrin is also involved in neutrophil migration into tumors (123). A study examining the recruitment of CD11bpos myeloid cells in response to tissue damage and hypoxia in irradiated tumors corroborated the importance of CD18 in myeloid recruitment, whereas CD11b was redundant (124). Together this supports that neutrophil interaction with the vasculature are predominantly regulated by LFA-1-ICAM-1 interactions (**Figure 6**).

IL-17, the signature cytokine expressed by γδ T Cells and Th17 cells, was recently associated with shaping neutrophil recruitment to tumors (125, 126). The combined activation of endothelial cells by TNF and IL-17 synergistically increased P-selectin, E-selectin, and ICAM-1, which enhanced neutrophil specific rolling and adhesion to the vasculature. TNF in combination with IL-17 stimulation dampened VCAM-1 levels compared to TNF alone, whereas ICAM-1 levels remained highly expressed on the endothelium (90). Importantly, IL-17 and TNF synergistically increased the neutrophil-recruiting chemokines CXCL1 (GRO-α), CXCL2 (MIP2α), CXCL5 (LIX), and induced secretion of GM-CSF and G-CSF to mobilize myeloid cells including neutrophils from the bone marrow into the circulation (90, 127). Another study showed that Ly6Clow monocytes recruited Ly6G+ neutrophils *via* CXCL5 (128).

The anti-tumor functions of neutrophils are controversial, and substantial evidence suggest that the anti-tumor activity of neutrophils is context dependent. In pancreatic ductal adenocarcinoma the CXCR2-CXCL5 axis increased infiltration of neutrophils, which promoted tumor growth (128). Conversely, in esophageal squamous cell carcinoma IL-17 stimulated CXCL2 production by tumor cells, which is also associated with increased infiltration of neutrophils. However, in this context neutrophil infiltration induced anti-tumor effects since IL-17 potentiated the direct killing capability of neutrophils by enhancing the production of cytotoxic molecules, including reactive oxygen species (ROS), MPO, TNF-related apoptosis-inducing ligand (TRAIL), and IFNγ (129). Another example of beneficial anti-tumor neutrophils has been shown in the context of early stage epithelial carcinogenesis. Hypoxia induced expression of CXCL1 (GRO-α), CXCL2 (MIP2α), CXCL5 (LIX) recruited anti-tumor neutrophils, which inhibited tumor growth by inducing tumor cell detachment from the basement membrane (130). Neutrophils have also been implicated to induce vascular damage, resulting in ischemic hemorrhagic necrosis and tumor rejection, for instance in response to intratumoral IL-12 injections (131). This type of neutrophil mediated tumor death seemed to play a crucial role in the rejection of subcutaneous tumors but not in lung metastasis (131). Furthermore, DC101 (anti-VEGFR2) enhanced neutrophil and macrophage infiltration, and in combination with a glucuronide prodrug had increased anti-tumor activity in experimental mouse models (132). In a co-culture of UKF-NB-4 neuroblastoma tumor cells and human umbilical vein endothelial cells (HUVEC) it has been shown that direct tumor-endothelial cell-cell contact promotes downregulation of endothelial CD44 receptor expression, impairing neutrophil binding (133, 134). How endothelial CD44 inhibition may repel an effective anticancer attack by neutrophils was not investigated.

### Monocyte Trafficking Across Cancer Endothelium

Two monocyte type subsets have been identified, i.e., classical monocytes (Ly6Chi CD14pos CD16neg) and non-classical monocytes (Ly6Clow CD14neg CD16pos) (135). Both subsets show anti-tumor as well as pro-tumor effects, which are context-dependent (135). Classical monocytes mainly support tumor growth and metastasis through VEGF-A release, which promotes angiogenesis (136–138) and worsens clinical outcome (139). Classical monocytes have also been associated with anti-tumor effects (138, 140).

Classical monocytes express high levels of the chemokine receptor, CCR2 (136). CCR2 mediates monocyte activation upon recognition of immobilized CCL2 at the endothelium (136), which is mediated by TNF (141). This CCR2-CCL2 axis has shown to recruit angiogenic classical monocytes in PyMT spontaneous breast carcinoma, KCKO pancreatic carcinoma, and MC38 colorectal carcinoma (136, 139, 142, 143) (**Figure 7**). In a model for pancreatic carcinoma and liver metastasis CCR2 deficiency or CCR2 blockade has shown to impair classical monocyte recruitment, angiogenesis inhibition and relief of immunosuppression (139, 143). In addition, CCR2 inhibition decreased the number of tumor associated MDSCs and improved anti-PD-1 therapy in resistant murine gliomas (144).

The use of E- and L-selectin-deficient mice in a lung cancer model (LLC) and CRC model (MC-38) has shown that selectin-mediated recruitment of monocytes and macrophages supports tumor metastasis (121, 122, 145). This indicates the importance of E-selectin (ligand for, e.g., PSGL-1) and L-selectin (ligand for, e.g., GlyCAM-1, CD34, etc.) for myeloid cell recruitment in cancer (62).The integrin VLA-4 has been shown to regulate recruitment of classical monocytes to tumors promoting angiogenesis (146). VLA-4 is known to interact with VCAM-1. However in the context of vascular abnormalities in cancer, monocytes may use VLA-4 to bind to fibronectin, as the disruptive endothelium in cancer contains many intercellular gaps, hereby exposing fibronectin (**Figure 7**). Indeed, VLA-4 has shown to be important for selective homing of monocytes to tumors (146).

An interesting phenomenon affecting leukocyte trafficking and immunosuppression in cancer involves the effects of hypoxia on myeloid cells. Hypoxia causes myeloid cells to produce arginase (ARG) and nitric oxide synthase. Arginase inhibits effector T cell function inducing immunosuppression (119, 147). Moreover, nitric oxide synthase is an important producer of peroxynitrate, a molecule important to form reactive nitrogen species (RNS), affecting selective trafficking of leukocytes into tumors. RNS have been implicated in chemokine nitrosylation, a posttranslational modification forming chemical barriers for certain leukocyte subsets. Nitrosylated CCL2 forms a physical barrier for cytotoxic T cells and Th1 effector cells (**Figure 7**). Conversely, nitro-CCL2 recruits MDSCs supporting angiogenesis and tumor growth (13). CCR2 knockout mice fail to recruit MDSC to cancers (148). High levels of CCL2 have been found in breast, gastric and ovarian cancers, which correlated with MDSC infiltration (148).

In contrast to CCR2-dependent recruitment of classical monocytes, the mobilization and homing of non-classical monocytes (also known as patrolling monocytes) to tumors involves the chemokine receptor CX3CR1 (149) (**Figure 7**). CX3CR1 mediates the migration of non-classical monocytes through CX3CL1 recognition on cancer endothelium. In melanoma, breast and lung cancer non-classical monocytes show anti-tumor effects predominantly through recruitment of cytotoxic NK cells (150–153). Conversely, in colorectal cancer, non-classical monocytes promoted tumor growth through induction of angiogenesis and immune suppression (51, 52, 154). Interestingly, these non-classical monocytes also release CXCL5 to recruit neutrophils and produce IL-10, inhibiting adaptive immunity (52).

## Tumor Dissemination *via* Vascular and Lymphatic Networks

Pathological angiogenesis in cancer forms an abnormal tumor vascular network. The connection of this abnormal vascular network to the vascular system provides a conduit for cancer cells to spread over the body, facilitating tumor metastasis (155, 156). Abnormal tumor vessels are characterized by an immature morphology, meaning lower pericyte and smooth muscle cell coverage, discontinuous endothelial cell-cell junctions, and an abnormal basement membrane (157, 158). Moreover, the blood flow in tumors does not always follow a constant unidirectional path, and an extensive amount of the tortuous tumor vascular network is not well perfused (46, 159). Despite less perfusion of tumor vessels metastasis *via* the blood circulation occurs more frequently than distant metastasis *via* the lymphatics (155). Pericytes are important supportive stromal cells for endothelial cell function and monolayer integrity. Consequently, the lack of pericyte coverage cause improper and loose endothelial cell-cell contacts, leaky, and permeable vessels which increase the number of tumor cells to metastasize *via* the blood stream (158). Dysfunctional angiogenic vasculature in tumors generally increases hypoxia, which can further promote cancer cell motility and metastasis through the activation of epithelial to mesenchymal transition (EMT) (160, 161). EMT mechanisms decrease epithelial cell-to-cell junctions and increase cancer cell motility, enhancing invasiveness and cancer escape. Downregulation of E-cadherin, an important molecule for stability of cell-cell contacts, and upregulation of the transcriptional factors Snail1, Snail2, Slug, and Twist are described as main drivers for EMT (161, 162). In addition, hypoxia related TGF-β3 induction (163, 164) and Notch signaling pathways are reported as EMT promoting mechanisms as well (165).

In addition to EMT mechanisms and metastasis *via* the blood circulation, abnormal lymphatic vessels and lymph angiogenesis also facilitate tumor dissemination. VEGF-C and VEGF-D are key promoters of tumor-associated lymph angiogenesis, increasing lymphatic vessel diameter (166) and cancer metastasis *via* lymphatics (166–169). Blocking VEGF-C and VEGF-D signaling with neutralizing antibodies or soluble VEGFR3 correlated with suppressed tumor lymph angiogenesis and lymph node metastasis (170, 171). Moreover, the leaky nature of vessels combined with dysfunctional lymphatic drainage results in increased interstitial fluid pressure in the tumor microenvironment. Consequently, high fluid pressure drives cancer cells passively into enlarged tumor associated lymphatic vessels and supports cancer metastases to distal organs (172, 173). An active mechanism of tumor dissemination *via* lymphatics involves the chemokines CCL21 and CCL19, which are secreted by tumor cells. In response to high fluid pressure CCL21 and CCL19 are pushed towards tumor draining lymph nodes, which are simply followed by cancer cells that sense the fluid pressure induced chemokine gradients *via* CCR7 (166). Altogether, the vascular network in tumors sustains a favorable niche for cancer cells to thrive and ultimately metastasize.

## A RationalE to Combine Immunotherapy With Anti-Angiogenic Agents

Vascular abnormalities are a hallmark of most solid tumors. Targeting angiogenesis in tumors with monoclonal antibodies such as Bevacizumab (anti-VEGF) were initially aimed to starve tumors to death and prevent metastatic spread. However, the benefits of Bevacizumab used as a single agent were modest evidenced by limited tumor regression and low overall survival (174–176). The lack of durable anti-tumor responses might be attributed, in part, by compensatory mechanisms in angiogenesis pathways, including alternative angiogenesis pathways such as the Ang2 pathway (177), co-option of nearby vessels or vessel mimicry (73, 178). Moreover, tumors that relapsed from angiogenesis treatment showed increased expression of immune inhibitory checkpoints such as PD-L1 (179), which provides a rational to combine anti-angiogenic agents with checkpoint blockade.

As outlined, pro-angiogenic proteins have profound effects on a broad range of immune cells, which alter immune cell maturation, activity and trafficking and hereby support immune suppression and tumor immune evasion. A logical intervention to tackle the leukocyte trafficking problem and immunosuppression in cancer is to also target angiogenic tumor vessels in addition to immunotherapy. The majority of the anti-angiogenic approaches (e.g., anti-VEGF antibodies or VEGF tyrosine kinase inhibitors (TKI)) induce vessel normalization, which is characterized by increased vessel perfusion, pericyte coverage (180), increased oxygen (181–183), reduced permeability (36), regression of tortuous vasculature (184–186), reduced interstitial fluid pressure (185), and upregulation of Ang-1 gene expression (184). In addition, targeting pro-angiogenic proteins such as VEGF counteracts immunosuppressive effects on immune cells as a result of reduced hypoxia in tumors (15, 17, 37). Furthermore, substantial evidence underscores the potential of angiogenesis inhibition to restore endothelial function allowing the infiltration of cytotoxic immune cells by improving vascular-leukocyte interactions and chemotaxis (9, 31–34, 187, 188) which overall may improve the efficacy of many immunotherapy formats.

Likewise, preclinical studies show that immune checkpoint blockade brings about anti-vascular immune responses against tumor vessels showing the synergistic potential of combining anti-angiogenic agents with immunotherapy (179, 189–191).

Anti-angiogenic agents yield a therapeutic window of vascular normalization in which new strategies can be explored to increase infiltration of cytotoxic immune cells improving the efficacy of immunotherapy. This may yield a better overall efficacy. The dosing of anti-angiogenic agents, however, needs to be carefully evaluated as high dose can also result in increased vessel pruning and hypoxia, mediating resistance to therapy (37, 73, 177). Altogether, anti-angiogenic agents have the potential to reprogram the immunosuppressive TME to a microenvironment that supports anti-tumor immunity.

## Targeting Tumor Vessels to Enhance Leukocyte Trafficking and Immunotherapy

**Figure 8:**
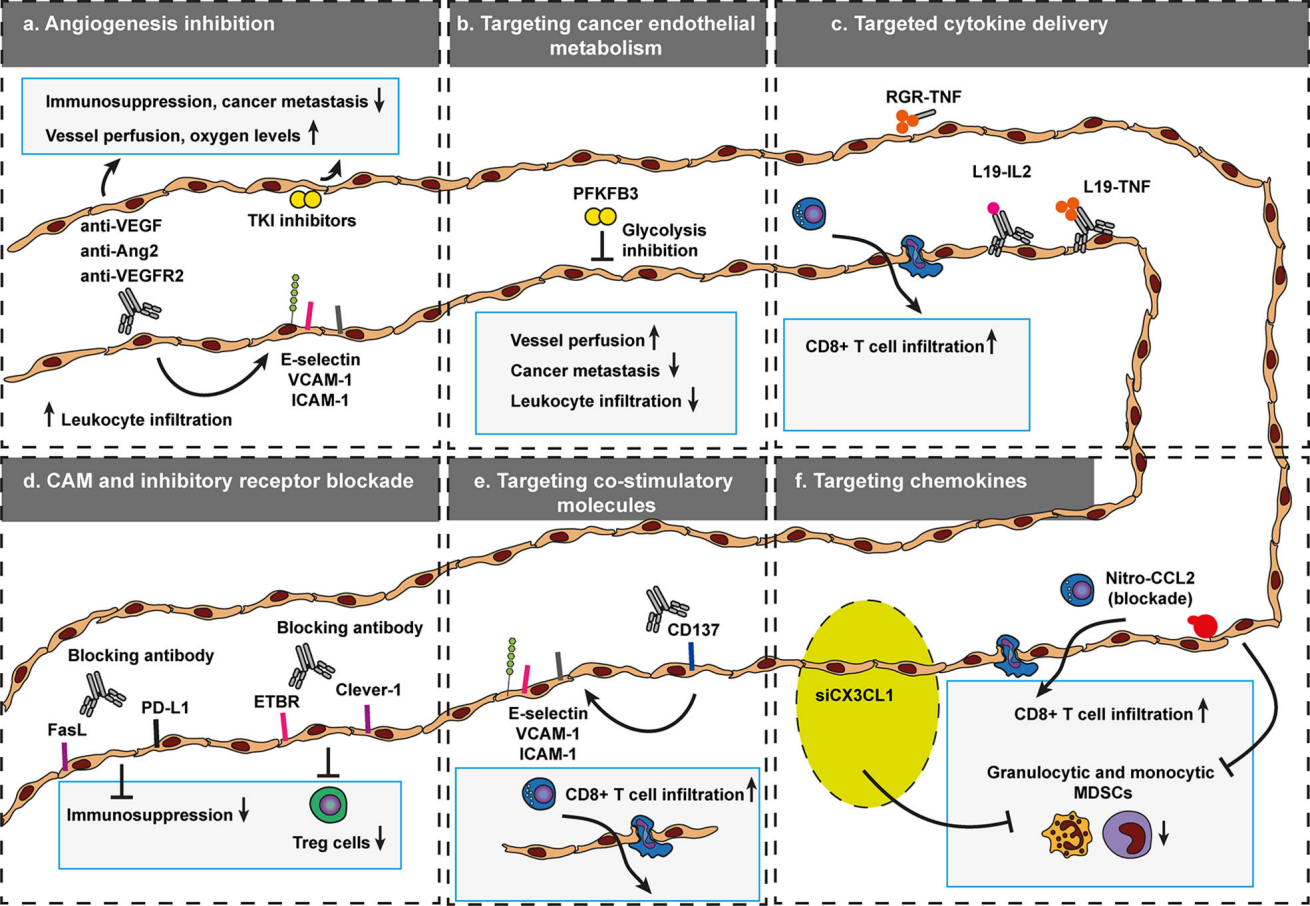
Targeted approaches that govern selective trafficking of leukocytes across cancer endothelium. To data, a variety of vascular targeting approaches have been developed to modulate leukocyte trafficking to increase the efficacy of anti-cancer therapy. These include inhibition of angiogenesis **(A)**, targeting cancer endothelial metabolism **(B)**, targeted cytokine delivery **(C)**, CAM, and immune inhibitory receptor blockade **(D)**, targeting co-stimulatory molecules **(E)**, and silencing of leukocyte recruiting chemokines **(F)**. Targeting agents that affect selective trafficking of leukocytes across cancer endothelium become increasingly relevant as the success of many immunotherapy formats also depends on effective recruitment of cytotoxic or inhibition of immunosuppressive immune cells. Endothelin B receptor (ETBR), common lymphatic endothelial, and vascular endothelial receptor-1 (Clever-1, also known as stabilin-1). Programmed death-ligand 1 (PD-L1), Fas ligand (FasL).

To date, there are a handful of approaches to increase selective trafficking of leukocytes into tumors. These include anti-angiogenic agents, blocking antibodies targeting key surface molecules involved in leukocyte traffic, targeted delivery of pro-inflammatory cytokines and nanoparticles that deliver content to silence leukocyte specific chemokine expression (**Figure 8**). Because tumor vasculature is very distinct from resting endothelial cells in healthy tissue, a great variety of surface molecules, commonly involving angiogenesis related proteins, are available to enable targeted therapy of tumor vessels.

### Anti-Angiogenic Approaches

An established way of targeting tumors and increase leukocyte infiltration in tumors is through inhibition of angiogenesis (**Figure 8A**) including anti-VEGF/VEGFR2 (32–34, 179, 187, 192–195), Ang2 (190, 196), VEGF TKI (9, 31, 188, 197, 198), or Tie2 agonists (36). The majority of these anti-angiogenic approaches induce vessel normalization, which is characterized by increased vessel perfusion, pericyte coverage (180), increased oxygen (181–183), reduced permeability (36), regression of tortuous vasculature (184–186), increased leukocyte traffic (9, 31–34, 187, 188) reduced interstitial fluid pressure (185) and upregulation of Ang-1 gene expression (184).

Vascular normalization creates a window of opportunity to combine anti-angiogenic agents with other anti-cancer therapies, especially with immunotherapy (15, 17). As such, targeting tumor vessels with anti-angiogenic agents in combination with immune inhibitory checkpoints (9, 179, 190, 195, 196, 199–202), adoptive cell transfer (34, 187, 193, 194, 197, 203), or cancer vaccines (31–33, 188, 192, 198) has shown improved anti-cancer effects. Normalization effects of anti-VEGF on tumor vasculature can already observed as early as one day after treatment in mice and human, as more matured vessels were observed as well as reduced tortuous vessels, which had smaller diameter and were shorter in length (204). The time that the therapeutic window persists depends on the therapy dosage (32, 174) and on tumor origin (19). At the right dose, the window might last for about a week in mice (32, 204). In humans the window is suggested to last longer (15, 19).

Perhaps counterintuitive, extensive treatment, and high dose anti-angiogenic agents shorten the therapeutic window as this leads to enhanced vessel pruning, which leads to vascular disruption and increased hypoxia that may ultimately result in induction of alternative angiogenesis pathways such as Ang2 pathway, hereby driving therapeutic resistance (73, 177). This also suggests that targeting multiple angiogenic pathways simultaneously may enhance angiogenic therapeutic responses. Combined targeting of Ang2 and VEGF pathways was show to extend the normalization window (35, 205, 206).

In addition to the effects of VEGF blockade on the tumor vasculature, single targeting of VEGF or in combination with Ang2 blockade converted the immunosuppressive environment into an immune stimulatory environment, evidenced by increased detection of M1 macrophages and increased CD8+ T cells infiltration in GL261 glioblastoma tumors (35), or in a model of MCaP0008 breast cancer (32). Furthermore, inhibition of angiogenesis and simultaneously activating the Tie2 pathway (stabilizing/strengthening endothelial junctions) normalized vessels effectively, increasing oxygen levels and diminished immunosuppression (36). Because the majority of anti-angiogenic approaches relieve immunosuppression (32, 35) and increase leukocyte entry into tumors through normalization of tumor vessels (32), the use of anti-angiogenic therapy in combination with immunotherapy exhibits great potential to enhance tumor killing.

The potential to combine anti-angiogenic therapy with immunotherapy, in particular with checkpoint inhibitors, was corroborated by many combinations in various preclinical mouse models (**Table 1**) and tested in a large number of clinical phase I, II and III trials that confirm the synergistic effect of combinational therapy (**Table 2**). In pre-clinical studies the general outcome of combining anti-angiogenic agents with immunotherapy shows increased tumor regression and overall survival, often associated with relief of immunosuppression, evidenced by reduced presence of tumor resident MDSC and Treg cells, which often coincided with increased infiltration of CD4 and CD8 T cells (9, 31–34, 187, 188, 221). However, the majority of studies do not include metrics to show that vascular normalization is achieved. Blocking anti-VEGF in combination with checkpoint inhibition and its effects on the vasculature should be investigated in more detail in further research.

**Table 1:** Preclinical studies on combinations of antiangiogenic agents and immunotherapies.

Immunotherapy	Antiangiogenic Therapy	Disease	Key Results	Refs
***Vaccination Studies***				
Tumor-antigen-specific picornaviral vaccination	Aflibercept (anti-VEGF)	Glioblastoma	CD8+ T cells ↑Tumor progression ↓ and animal survival ↑	(192)
Whole tumor cell vaccine (mitomycin treated and GM‑CSF secreting)	DC101 (anti-mouse VEGFR2 mAb)	Breast cancer	CD4+ and CD8+ T cells ↑MDSCs and Treg cells ↓Tumor regression and animal survival ↑	(32, 33)
Pox virus vaccine expressing CEA and three co‑stimulatory molecules	Sorafenib (VEGFR-TKI)	Colon cancer	Intratumoral CD8+ T cells ↑ and Treg cells ↓MDSCs and Treg cells ↓Tumor volume ↓Animal survival ↑	(198)
OVA peptide-pulsed DC (VAC)	Sunitinib (TKI)	Melanoma	Anti-tumor efficacyrecruitment of Type-1 anti-tumor T cells into the TME ↑MDSC ↓Treg ↓	(31)
MVA/rF-CEA/TRICOM	Cabozantinib (TKI)	Colon cancer	MDSCsf and Treg cellsf ↓CD4-positive and CD8-positive T-cellinfiltration ↑	(188)
***Cell Therapy Studies***				
Anti‑PMEL T cells, PMEL vaccine, and IL‑2	DC101 (anti‑VEGFR2 mAb) and B20 (anti-VEGF mAb)	Melanoma	Immune cell infiltration ↑Tumor growth ↓Animal survival ↑	(34)
Anti‑VEGFR1 chimeric antigen receptor T cells		Lung cancer	Endothelial tube formation *in vitro* ↓Tumor growth and metastasis ↓	(194)
Tumor-antigen-specific CD8+ T cell transfer	Sunitinib (TKI)	HCC	Complete tumor regression	(197)
GM-CSF–secreting tumor cell immunotherapy	sVEGFR1/R2	Melanoma, Colon cancer	Animal Survival ↑CD4+ and CD8+ tumor-infiltrating T cells ↑Treg cellsf ↓	(165)
***Immune Checkpoint Inhibitors***				
Anti‑PD‑1 mAb	Vanucizumab (Anti-Ang2 Anti-VEGFA),	Breast cancer, Melanoma, Pancreatic cancer, Neuroendocrine cancer	Tumor growth ↓Animal survival ↑	(190)
Anti‑PD‑1 mAb	DC101 (anti‑VEGFR2 mAb)	Colon cancer	Angiogenesis ↓T cell infiltration ↑Cytokine expression ↑	(195)
Anti‑PD‑1 mAb	Sunitinib (TKI)	Colon cancer	PD‑1+CD8+ T cells ↓Anticancer activity ↑	(9)
Anti‑PD‑L1 mAb	DC101 (anti‑VEGFR2 mAb)	Pancreatic cancer, Breast cancer, Glioblastoma	IFNγ-expressing CD8+ and IFNγ-expressing CD4+ T cells ↑PD‑L1 expression on relapsing tumor cells ↑Vessel normalization ↑ by PD‑L1 blockade and formation of HEVs ↑	(179)
Anti‑PD‑L1 antibody	CVX‑060 (ANG2‑specific peptide–antibody fusion protein) ± sunitinib or regorafenib (both VEFGR TKIs) or CVX‑241 (bi‑specific ANG2–VEGF-binding peptide–antibody fusion protein)	Breast cancer, Colorectal cancer, Renal cancer	Tumor growth and metastatic progression ↓ with combined inhibition of ANG2 and VEGF signalling (with or without immune-checkpoint blockade)	(196)
Anti-mouse CTLA-4 antibody	Axitinib (Anti-VEGFR)	Melanoma	Effector T cell ↑ Animal Survival ↑	(199)
Anti‑PD‑L1 antibody	Anti-VEGF	Small-cell lung cancer (SCLC)	Animal Survival ↑CD4+ T-cell infiltration ↑	(200)

**Table 2:** Clinical studies on combinations of antiangiogenic agents and immunotherapies.

Immunotherapy	Antiangiogenic therapy		Disease	Status	ClinicalTrials.gov identifier (publication)
***Vaccination Studies***					
PF 06755990 (vaccine)		Sunitinib (TKI)	Prostate cancer	Phase I:	NCT02616185
Pox virus vaccine (expressing GM CSF)		Sorafenib (TKI)	HCC	Phase III:	NCT02562755
***Immunostimulatory Cytokine Studies***					
IFNα		Bevacizumab (anti-VEGF)	Metastatic RCC	Phase III (CALGB 90206): completed	NA (207)
IFNα2A		Bevacizumab (anti-VEGF)	Metastatic RCC	Phase III (AVOREN): completed	NA (208)
***Cell Therapy Studies***					
NK cell-based immunotherapy		Bevacizumab (anti-VEGF)	Advanced-stage solid tumors	Phase I/II: completed	NCT02857920
Autologous DC immunotherapy		Sunitinib (TKI)	Advanced-stage RCC	Phase II: completed	NCT00678119 (209)
***Immune-Checkpoint Blockade Studies***					
Ipilimumab (anti-CTLA-4)		Bevacizumab (anti-VEGF)	Advanced-stage melanoma	Phase I: completed	NA (210)
Ipilimumab (anti-CTLA-4) or Nivolumab (anti-PD-1)		Bevacizumab (anti-VEGF)	Metastatic melanoma	Phase NA: completed	NA (201)
Ipilimumab (anti-CTLA-4)		Bevacizumab (anti-VEGF)	Metastatic RCC	Phase I:	NCT02210117
Ipilimumab (anti-CTLA-4)		Bevacizumab (anti-VEGF)	Melanoma	Phase II:	NCT01950390
Ipilimumab (anti-CTLA-4)		Bevacizumab (anti-VEGF)	Melanoma	Phase I:	NCT00790010
Nivolumab (anti-PD-1)		Bevacizumab (anti-VEGF)	NSCLC	Phase I: (safety and tolerability, ORR, and RFS)	NCT01454102
Nivolumab (anti-PD-1)		Sunitinib or pazopanib (TKI)	Metastatic RCC	Phase I:	NCT01472081 (211)
Pembrolizumab (anti-PD-1)		Axitinib (TKI)	RCC	Phase III:	NCT02853331
Pembrolizumab (anti-PD-1)		Nintedanib (broad TKI and nTKI)	Advanced-stage solid tumors	Phase I:	NCT02856425
Pembrolizumab (anti-PD-1)		Bevacizumab (anti-VEGF)	Glioblastoma	Phase II: completed	NCT02337491
Pembrolizumab (anti-PD-1)		Ramucirumab (anti-VEGFR2)	Gastric or gastro-oesophageal adenocarcinoma, NSCLC, urothelial carcinoma, or biliary tract cancer	Phase I: completed	NCT02443324 (212)
Atezolizumab (anti-PD-L1)		Bevacizumab (anti-VEGF)	Non-clear-cell RCC	Phase II:	NCT02724878 (213)
Atezolizumab (anti-PD-L1)		Bevacizumab (anti-VEGF)	Metastatic cervical cancer	Phase II:	NCT02921269
Atezolizumab (anti-PD-L1)		Bevacizumab (anti-VEGF)	Multiple solid tumors	Phase I:	NCT01633970 (214)
Atezolizumab (anti-PD-L1)		Bevacizumab (anti-VEGF)	Non-Small-Cell Lung Cancer	Phase III:	NCT02366143 (215, 216)
Atezolizumab (anti-PD-L1)		Vanucizumab (bi‑specific mAb targeting VEGF and ANG2)	Advanced-stage solid tumors	Phase I: completed (MTD, AEs, and ORR)	NCT01688206
Avelumab (anti-PD-L1)		Axitinib (TKI)	Advanced-stage RCC	Phase I:	NCT02493751 (217)
Avelumab (anti-PD-L1)		Axitinib (TKI)	Advanced-stage RCC	Phase III:	NCT02684006 (218, 219)
Selicrelumab (RO7009789-agonistic anti‑CD40 mAb)		Vanucizumab (bi‑specific mAb targeting VEGF and ANG2)	Metastatic solid tumors	Phase I: completed (safety, pharmacokinetics and pharmacodynamics, and therapeutic activity)	NCT02665416
Durvalumab (anti-PD-L1)		Ramucirumab (anti-VEGFR2)	Gastric or gastro-oesophageal adenocarcinoma, NSCLC or HCC	Phase I:	NCT02572687
SHR‑1210 (anti‑PD‑1 mAb)		Apatinib (TKI, VEGFR2)	Gastric cancer and HCC	Phase I/II: (tumor control rate, disease control rate, OS, and AEs)	NCT02942329 (220)

Several clinical trials corroborated the increased progression-free survival rates found in preclinical studies. In a phase III trial, investigating the survival benefits for patients with advanced renal-cell carcinoma upon dual targeting with checkpoint inhibitors and angiogenesis blockade, combined effects of pembrolizumab (anti-PD-1) with axitinib (VEGFR TKI) resulted in a significantly longer overall and -progression-free survival, as well as a higher objective response rate, compared to sunitinib (VEGFR TKI) treatment alone (222). Another study (phase I), tested the combined effects of the immune checkpoint CTLA-4 (ipilimumab) and VEGF inhibition by bevacizumab on immune infiltration in metastatic melanoma patients. This study corroborated the effect of antiangiogenic agents on vessel normalization and activation, evidenced by increased E-selectin expression on intratumoral endothelial cells, which coincided with increased infiltration of CD8^+^ cytotoxic T cells, CD163^+^ macrophages and minimal change in Foxp3^+^ Treg cells (210). Moreover, a study combining bevacizumab and an anti-tumor vaccine, followed by adoptive T-cell transfer showed clinical benefit for recurrent ovarian cancer, including one complete response (223). Together, this data emphasizes the power to combine anti-angiogenic therapy with immunotherapy and highlight its clinical potential.

### Targeting Cancer Endothelial Metabolism

In addition to the more established vascular normalizing-agents anti-VEGF or -ANG2, an emerging way of inducing vascular normalization is introduced through targeting of endothelial cell metabolism (**Figure 8B**). Endothelial sprouting involved in angiogenesis relies on glycolysis rather than oxidative phosphorylation for ATP production. As such blocking PFKFB3, a key molecule involved in endothelial glycolysis, reduced vessel sprouting and angiogenesis (224, 225). In a cancer setting, transient reduction of glycolysis by PFKFB3 blockade improved vessel normalization, impaired tumor metastasis and improved chemotherapy delivery (226). In this setting, vessel normalization was characterized as a tighter barrier with increased pericyte coverage. However, glycolysis blockade also interfered with NF-κB signaling, which reduced endothelial adhesion molecules, i.e., ICAM-1 and VCAM-1. The reduction of CAM expression reduced metastatic spread, but might also interfere with infiltration of cytotoxic effector cells. In addition, there are also dose dependent limitations of using PFKFB3 blockade. High dose PFKFB3 aggravates vessel integrity, tumor hypoxia, and metastasis, highlighting the importance of adequately dosing a glycolytic inhibitor for anticancer treatment (227). As such, future research should identify whether endothelial glycolysis blockade in combination with immunotherapy is beneficial. Moreover, further studies now investigate the potential of studying tumor endothelial cell metabolism and its heterogeneity (228).

## Modulating Selective Leukocyte Recruitment During Vascular Normalization

Vascular normalization with anti-angiogenic drugs or glycolysis blockade may create a therapeutic window for immunotherapy, but success of therapeutic efficacy also depends on either effective recruitment of cytotoxic immune cells or inhibition of immunosuppressive cells. Therefore, targeting agents that affect selective trafficking of leukocytes across cancer endothelium become increasingly relevant. Thus far, a couple of approaches have been developed to regulate immune cell trafficking to provide access and selectivity for some immune cell subtypes to cross the endothelial barrier (**Figures 8C–F**).

First, selective immune cell trafficking was regulated through targeted-delivery of cytokines using cytokine-peptide fusions or cytokines-antibody fusion, i.e., immunocytokines (**Figure 8C**). An promising example is the targeted delivery of low dose TNF using a TNF-RGR peptide, which increased vessel stabilization, vessel perfusion and enhanced T cell infiltration, hereby improving overall survival after antitumor vaccination or adoptive T-cell therapy (229). Similar results were obtained with a NGR-TNF peptide (230). Another method involves immunocytokines, of which the target molecules are often angiogenesis related molecules in solid cancers, including EDA and EDB domains of fibronectin and the extra-domain A1 of tenascin-C. These molecules are specifically expressed in angiogenic parts of tumor vasculature but not detectable in normal tissues (231). F8, L19 and F16 antibodies are specific to the EDA, EDB, and A1 domain, respectively (232, 233). As such, these antibodies are ideal to generate cytokine-fusion antibodies targeting tumor associated vasculature.

L19-IL2 is a well described immunocytokine having a central role in the regulation of T cell responses and effects on other immune cells, such as natural killer cells, B cells, monocytes, macrophages and neutrophils. IL2 can induce tumor regression through its ability to stimulate a potent cell-mediated immune response *in vivo* (234, 235). L19-IL2 as well as L19TNF are now being evaluated in phase II clinical trials through intratumoral application in stage III or IV melanoma patients (236).

Targeting tumor vasculature with anti-angiogenesis agents or immunocytokines increases leukocyte trafficking through upregulation of CAM expression on cancer endothelium. As such, blocking key adhesion receptors involved in leukocyte transendothelial migration might be an effective approach to modulate selective trafficking of leukocytes (**Figure 8D**). Although generic CAMs can bind integrins expressed by several leukocyte subtypes, blocking specific CAMs might govern selective migration across tumor vessels. For instance, blocking antibodies targeting Clever-1 or ETBR, which are key surface molecules involved in Treg cell transmigration across tumor vasculature, impaired Treg cell but not CD8+ T cell entry into tumors (10, 103, 108).

Another approach to modulate selectivity through endothelial CAM expression is targeting the co-stimulatory molecule CD137 (4-1BB), which is selectively expressed on the surface of tumor endothelial cells, and induced by hypoxia. Agonistic antibodies targeting CD137 increase E-selectin, ICAM-1, and VCAM-1 surface expression on tumor vessels. This restoration of CAM expression increased T cell trafficking across tumor blood vessels improving infiltration of T lymphocytes into malignant tissue (237) (**Figure 8E**).

Finally, selective trafficking of leukocytes might be modulated through interfering with chemokine signals. Such an approach may involve targeted delivery of siRNAs that locally interferes with chemokine signaling affecting the selective trafficking of certain leukocytes into the TME (**Figure 8F**). Several lines of data show the feasibility of this approach. For instance, *in vivo* nanoparticle delivery of siCX3CL1 inhibited non classical Ly6C^lo^ monocyte infiltration and enhanced efficacy of anti-VEGFR2 therapy (154). Additionally, intelligent nanorobots have been described that are able to deliver anticancer therapeutics to tumor vessels, targeting tumor endothelial specific nucleolin (238). In this case thrombin was locally delivered to induce rapid shutdown of tumor vasculature, which induced necrosis and inhibition of tumor growth. Similar nanorobots may also deliver cytokines and/or anti-angiogenesis agents locally to tumors to redirect selective trafficking of leukocytes and to modulate the tumor microenvironment.

## Concluding Remarks and Future Directions

In conclusion, vascular normalization with anti-angiogenic agents may counteract immunosuppression and increase leukocyte entry into tumors, hereby opening a window of opportunity to combine anti-angiogenic agents with immunotherapy. Understanding the regulation of selective immune cell trafficking may establish better approaches that govern selective immune cell infiltration in cancer to make better use of the therapeutic window during vascularization normalization. So far, the majority of research is focused on increasing the infiltration of cytotoxic immune cells in tumors. However, the selective inhibition of immunosuppressive immune cells might be equally important, as these cells modulate the activity of cytotoxic immune cells after they have entered the TME. Thus, molecules involved in selective trafficking might provide novel predictive markers to provide a rational for combining vascular targeting agents with specific immunotherapy strategies. Ultimately, improving immunotherapy through vascular normalization may lead to significantly better clinical outcome of many cancer patients.

## Author Contributions

GD, ME, and NH co-wrote the manuscript. NH prepared the figures. All authors contributed to the article and approved the submitted version.

## Funding

NH is supported by the Dutch Cancer Society grant 12863 and Cancer Center Amsterdam grant CCA2017-5-39. ME is supported by the Dutch Cancer Society grant 12749. GD is supported by the Program of Selection and Placement of Students for Overseas Postgraduate Education (YLSY) scholarship, awarded by the Turkish Ministry of National Education.

## Conflict of Interest

The authors declare that the research was conducted in the absence of any commercial or financial relationships that could be construed as a potential conflict of interest.
